# SLIT3 deficiency promotes non-small cell lung cancer progression by modulating UBE2C/WNT signaling

**DOI:** 10.1515/biol-2022-0956

**Published:** 2024-10-29

**Authors:** Zidan Qiu, Ying Zhan, Zhiyong Chen, Wenjin Huang, Jianrong Liao, Zhen Chen, Junqiong Zheng, Qiuxiang Zheng, Cuiping Lu

**Affiliations:** Longyan First Affiliated Hospital of Fujian Medical University, Jiuyi North Road No. 105, Xinluo District, Longyan, 364000, Fujian, The People’s Republic of China

**Keywords:** NSCLC, SLIT3, prognosis, migration, SLIT3/UBE2C/WNT signaling

## Abstract

In our prior research, it was noted that slit guidance ligand 3 (SLIT3), a member of the SLIT-secreted protein family, may play a potential role in tumorigenesis. In addition, our prior work has found that the SLIT3 gene is highly methylated, especially in advanced-stage lung cancer tissues. Herein, we propose the hypothesis that abnormal SLIT3 expression may be linked to lung cancer development. In this study, decreased SLIT3 at the transcriptome and proteome levels was observed in lung cancer tissues. Furthermore, the downregulation of SLIT3 was related to a higher tumor stage and poorer prognosis. Silencing SLIT3 expression enhanced cell proliferation and migration, indicating potential characteristics of a tumor suppressor gene of SLIT3 in non–small-cell lung cancer (NSCLC). Furthermore, SLIT3 deficiency stimulates UBE2C upregulation and regulates NSCLC progression through Wnt3A/β-catenin signaling. The activation of the WNT signaling pathway was highly correlated with chemoresistance development in lung cancer. In conclusion, SLIT3 deficiency promotes lung cancer onset and progression by modulating UBE2C/WNT signaling. SLIT3/UBE2C/WNT may serve as novel biomarkers and therapeutic targets in NSCLC.

## Graphical abstract

**Figure:**
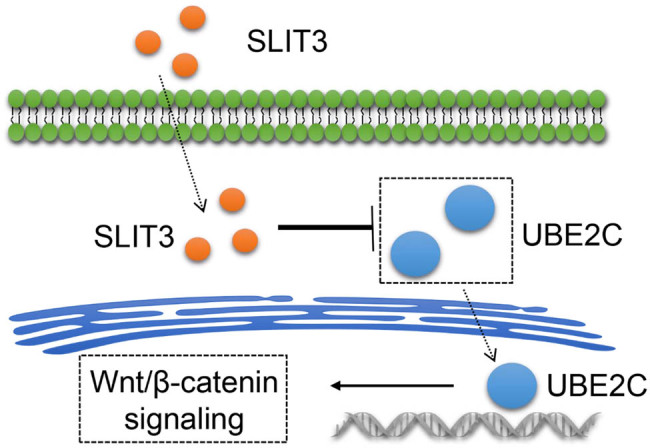

## 1 Introduction

According to the latest cancer statistics, there were an estimated 870,982 new cases of lung cancer in 2022, with 76,6898 death events, positioning it as the fifth most prevalent malignancy in China [1]. Non-small-cell lung cancer (NSCLC) is the most prevalent pathological type of lung cancer. Although multimodal systemic therapies have made tremendous advances, the treatment effect and outcome of NSCLC are still unsatisfactory, with a 5-year survival rate of less than 20% in late-stage populations [2]. As discussed elsewhere extensively, high tumor heterogeneity and complex molecular biological characteristics are key factors for the refractoriness and recurrence of NSCLC [3,4]. Thus, identifying the mechanisms that influence NSCLC tumorigenesis and progression would aid in developing novel therapeutic targets and improving the prognosis for NSCLC.

Numerous endogenous factors influence the initiation and progression of lung cancer, including tumor microenvironmental regulation, aberrant gene profiles, and oncogenic signaling pathways [5,6,7]. Among these factors, DNA methylation alterations are considered hallmarks of cancer [8]. Our prior work has found various hypermethylated genes in lung cancer tissues. Among them, the slit guidance ligand 3 (SLIT3) gene is highly methylated, especially in late-stage cancer tissues. Similar phenomena were also reported in other solid tumors. It was observed that dysregulated SLIT3 expression might affect tumor biology and behaviors, including tumor proliferation, migration, apoptosis, and tumor-related angiogenesis [9,10,11]. However, the function of SLIT3 in lung cancer is not well established yet. Herein, we propose the hypothesis that abnormal SLIT3 expression may relate to lung cancer development based on our previous findings.

Some studies have shown that SLITs were associated with tumorigenesis in breast cancer and liver cancer [12,13]. Therefore, the function of SLIT3 was further investigated in lung cancer. In certain tumors, limited evidence suggested that SLIT3 might be involved in the activation of cancer-promoting signaling pathways such as the ERK/MAPK and GSK3β/β-catenin signaling pathways [14,15]. However, the intricate details of this mechanism remain inadequately understood, particularly in the case of lung cancer. In lung cancer tissues, UBE2C, belonging to the E2 ubiquitin-conjugating enzyme family, has been found to have significantly increased expression compared with para-cancerous lung tissues. This finding implies the potential influence of this gene on disease prognosis and treatment [16]. Furthermore, UBE2C is intimately involved in a variety of signaling pathways in multiple cancers, including the WNT/β-catenin pathway [17]. In our prior work, it was observed that SLIT3 expression is highly correlated with UBE2C in NSCLC tissue samples, but the relationship between SLIT3 and UBE2C has not been identified yet. The roles of SLIT3 and UBE2C in the development and progression of lung cancer are still controversial.

Our research provided more evidence supporting the regulatory role of SLIT3 in driving aggressive biological behavior in NSCLC. Furthermore, this research elucidated the mechanism via which SLIT3 acts by regulating the UBE2C and WNT signaling. Collectively, SLIT3/UBE2C/WNT may act as novel biomarkers for NSCLC and hold promise as potential therapeutic targets in this disease.

## 2 Materials and methods

### 2.1 Cell culture and reagents

Human NSCLC cell lines (A549 and NCI-H1299) were acquired from the American Type Culture Collection (ATCC) and were cultured in PRMI-1640 medium containing 10% fetal bovine serum (FBS; Gibco, USA). The WNT signaling antagonist Wnt-C59 and chemotherapeutic reagents cisplatin (Cis) and paclitaxel (PTX) were provided by MedChemExpress (USA) and Sigma (USA), respectively. Paraffin sections from patients with NSCLC cancer were obtained from Longyan First Affiliated Hospital of Fujian Medical University (Ethics No. LYREC2023-k021-01).


**Informed consent:** Informed consent has been obtained from all individuals included in this study.
**Ethical approval:** The research related to human use has been complied with all the relevant national regulations, institutional policies and in accordance with the tenets of the Helsinki Declaration, and has been approved by the Ethics Committee of Longyan First Affiliated Hospital of Fujian Medical University (Ethics Approval No. LYREC2023-k021-01).

### 2.2 Cell proliferation assay

Cell Counting Kit-8 (CCK8) Assay Kit (Yeason, China) was employed for the detection of cell proliferation as per the instructions provided by the manufacturer. In a 96-well plate, cells were resuspended, seeded at a density of 2 × 10^3^ cells/well, and subsequently incubated at 37℃. The number of cells was recorded on a daily basis. The optical density of each sample was obtained at 450 nm with a microplate reader (Thermo Fisher, USA).

### 2.3 Transwell assay

A transwell migration assay was carried out to assess the migration potential of cells. The transwell insert (8 μm, Corning, USA) was utilized for seeding the treated A549 and NCI-H1299 cells (2.5 × 10^5^/ml). The upper chamber contained 100 μl of culture medium with 10% FBS and the bottom chamber contained 500 μl of culture medium with 20% FBS. After 24 h, 4% paraformaldehyde was used to fix the migrated cells on the bottom chamber, which were visualized with 1% crystal violet solution. The number of migrated cells was subsequently counted.

### 2.4 Cell apoptosis assay

Scramble or siRNA-treated tumor cells were cultured with Cis (1 μg/ml) or PTX (1 μg/ml) for 48 h. Subsequently, collected cells were washed with pre-cold PBS and resuspended at 2.5 × 10^5^/ml in the binding buffer supplemented with annexin V-FITC and PI (Becton, Dickinson and Company, USA). Cell apoptosis assays were performed by a flow cytometer (Becton, Dickinson, and Company, USA).

### 2.5 Patient information

Paraffin-embedded NSCLC sections were obtained from 20 patients diagnosed with NSCLC. These patients were divided into low-degree (stage III, LD) and high-degree (stage IV, HD) groups based on the TNM Classification of the WHO. Transcriptome expression data for SLIT3 and UBE2C, along with survival information, were obtained from the Sanger database, encompassing 513 NSCLC tissues and 397 normal tissues. In addition, we retrieved transcriptome expression and correlation analysis data for 230 NSCLC patients from the cBioPortal database.

### 2.6 Real-time quantitative polymerase chain reaction (qPCR)

Trizol reagent (Thermo Fisher, USA) was employed to isolate total RNA from lung cancer cells. Following this, the reverse transcription of total RNA into cDNA was carried out with a high-capacity cDNA Reverse Transcription Kit (Takara, Japan). Quantitative PCR analysis was conducted using a SYBR Green Kit (Takara, Japan). The primer sequences utilized in this research were as follows:

Slit3-Forward Primer: 5′- AGCGCCTTGACCTGGACA-3′;

Slit3-Reverse Primer: 5′-TCGGCGTGCTCTGGAAAA-3′;

UBE2C-Forward Primer: 5′-GGATTTCTGCCTTCCCTGAA-3′,

UBE2C-Reverse, 5′-GATAGCAGGGCGTGAGGAAC-3′;

WNT1-Forward Primer: 5′- GGGCACTGGACCATTAGATACA-3,

WNT1-Reverse Primer: 5′- TGAGTAGTCACAATACAGCCCT-3′;

WNT2-Forward Primer: 5′- GGGGCACGAGTGATCTGTG-3,

WNT2-Reverse Primer: 5′- GCATGATGTCTGGGTAACGCT-3;

WNT3A-Forward Primer: 5′- AGCTACCCGATCTGGTGGTC -3,

WNT3A-Reverse Primer: 5′- CAAACTCGATGTCCTCGCTAC-3′;

Actin-Forward Primer: 5′- CGAGCATCCCCCAAAGTT-3′’

Actin-Reverse Primer: 5′- GCACGAAGGCTCATCATT-3′;

### 2.7 RNA interference

A 24-well plate was utilized to seed the A549 or NCI-H1299 cells, which were incubated without serum for 4 h at 37°C, at a density of 2 × 10^5^ cells per well. To silence the expression of SLIT3 or UBE2C, cells were transfected with 50 nM of si-SLIT3 or si-UBE2C by using lipofectamine 3000 (Thermo Fisher, USA) for 48 h. The specific SLIT3 siRNA sequences were as follows: siRNA#1: 5′-GCAAAGAGCCGGUAGAUAUTT-3′ and siRNA#2: 5′-GUACAAAGAGCCAGGAAUATT-3′, and the specific UBE2C siRNA sequences were as follows: siRNA#1: 5′-GAAGTACCTGCAAGAAACCTACTCA-3 and siRNA#2: 5′-CAGCAGGAGCTGATGACCCTCATG-3′ (Tsingke Co, China). The negative control siRNA sequence was as follows: 5′-UUCUCCGAACGUGUCACGUTT-3′. The efficiency of silencing was assessed via qPCR reaction (SLIT3, siRNA #1, 67.4% and siRNA #2, 71.3%; UBE2C, siRNA#1, 59.8%, siRNA#2, 69.1%).

### 2.8 Western blotting

To verify the protein expression of SLIT3, UBE2C, and Wnt3A, anti-SLIT3 antibody (PA5-104142, Thermo Fisher, USA), anti–UBE2C antibody (MA1-173, Thermo Fisher, USA), anti-Wnt3A antibody (ab219412, Abcam, UK), and horseradish peroxidase-conjugated secondary antibodies were prepared for standard western blot analysis. In addition, 12% SDS-PAGE was utilized to separate the proteins, which were then blotted onto PVDF membranes after incubation with primary antibody and secondary antibody. The blots were visualized with enhanced chemiluminescence and exposed to hypersensitive chemiluminescence film.

### 2.9 Immunostaining

Lung cancer tissue sections were blocked for 30 min in a blocking buffer (5% bovine serum albumin) at room temperature. Samples were then immediately subjected to incubation overnight at 4°C with primary antibodies: anti-SLIT3 antibody (PA5-104142, Thermo Fisher, USA), anti-UBE2C antibody (MA1-173, Thermo Fisher, USA), and anti-Wnt3A antibody (ab219412, Abcam, UK). The next day, each sample underwent a brief incubation for 1 h at room temperature with horseradish peroxidase-conjugated secondary antibodies. The samples were visualized and analyzed under an optical microscope (Leica, Germany) or a confocal microscope (Olympus, Germany). Image-Pro Plus 5.1 software (MEDIA CYBERNETICS, USA) was employed to quantify the protein expression.

### 2.10 Animal protocols

Female NOD-SCID mice (6–8 weeks) were procured from Huafukang (Beijing, China) and raised in a clean environment. A total of 1 × 10^6^ A549 cells were implanted into the flanks of NOD-SCID mice (*n* = 5 in each group). Afterward, the mice underwent treatment with scramble or SLIT3 siRNA via intratumor injection on days 10, 15, and 20. Tumor volume and survival information were recorded daily. The calculation formula of tumor volume: tumor volume = length × width^2^/2. For the anticancer effect analysis of Wnt-C59, the mice treated with these siRNAs were subsequently administered PBS, PTX (10 mg/kg), Wnt-C59 (10 mg/kg), or a combination of them via tail vein injection on days 10, 15, and 20. All animal experiments adhered to ethical guidelines for animal research and were conducted according to the Helsinki Declaration.

### 2.11 Statistical analysis

GraphPad 6.0 software was utilized to analyze all experimental data. Data were entered as mean ± SD. The independent sample *t*-test was employed to determine the difference between the two groups. Furthermore, one-way ANOVA was utilized for the comparative assessment among multiple groups. Survival data were displayed by the Kaplan–Meier estimator. Each experiment was carried out at least three times independently. Statistical significance was considered at *p* < 0.05.

## 3 Results

### 3.1 SLIT3 deficiency-predicted poor prognosis in patients with NSCLC

**Figure 1:**
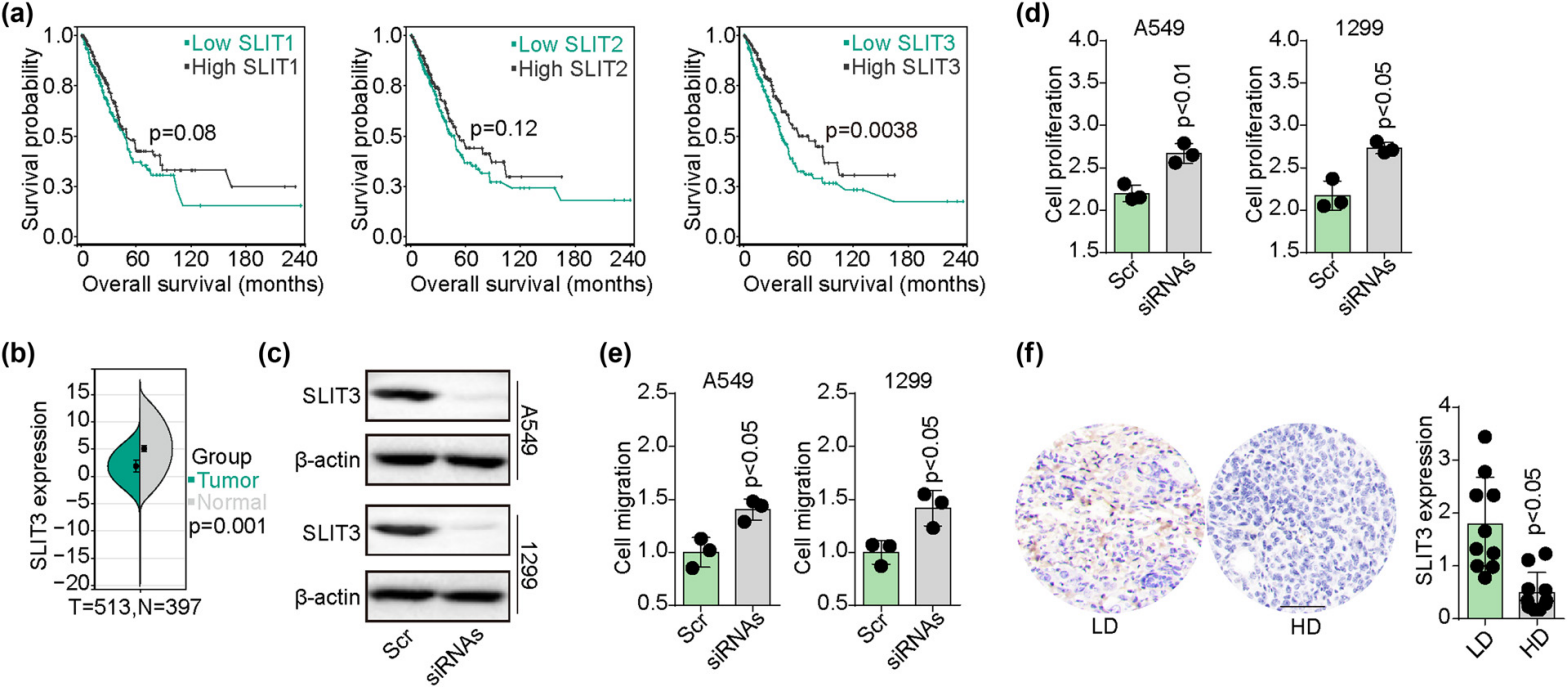
SLIT3 deficiency predicted poor prognosis in patients with NSCLC. (a) Kaplan-Meier overall survival curve was shown according to high and low expression of SLIT1∼3 in NSCLC patients (*n* = 513) derived from TCGA data. (b) mRNA expression of SLIT3 in 513 NSCLC tissues and 397 normal tissues derived from TCGA data. (c) Western blotting of SLIT3 in A549/NCI-H1299 cells treated with scramble and SLIT3 siRNAs. (d) Cell proliferation of A549/NCI-H1299 cells treated with scramble and SLIT3 siRNAs. (e) Cell migration of A549/NCI-H1299 cells treated with scramble and SLIT3 siRNAs. (f) Immunostaining of SLIT3 in NSCLC tissues from high-degree (HD, stage III–IV) and low-degree (LD, stage I–II) patients (*n* = 20). The scale bar was 100 μm.

To elucidate the clinical value of SLITs and their impact on the prognosis of patients with NSCLC, an analysis of the effect of SLITs (SLIT1–3) on overall survival was carried out in 531 NSCLC patients derived from The Cancer Genome Atlas (TCGA) database. It was observed that patients exhibiting low levels of SLIT3 had a shorter survival time compared to those with high levels of SLIT3 (Figure 1a). In addition, a comparison of SLIT3 transcriptome expression in 513 NSCLC tissues and 397 normal tissues from the TCGA database was carried out. The acquired data highlighted that SLIT3 was downregulated in NSCLC tissues compared to normal tissues (Figure 1b). Those results suggested that reduced SLIT3 may be indicative of a poor prognosis in patients with NSCLC. To further confirm the tumor suppressive effects of SLIT3, siRNA interference was utilized to silence the expression of SLIT3 in NSCLC cell lines A549 and NCI-H1299 (Figure 1c). The results showed that the silenced NSCLC cells displayed enhanced cell proliferative characteristics (Figure 1d) and migratory phenotypes (Figure 1e) compared to the scramble group. This observation suggested that cell proliferation and migration were suppressed by SLIT3 in NSCLC. Furthermore, the expression of SLIT3 was examined at the protein level through immunohistochemical staining in clinical specimens. Consistently, a remarkable decrease in its expression was noted in NSCLC tissues from patients with advanced disease (stage IV) compared to those with earlier stages (stage I–III, Figure 1f). Collectively, these findings indicate that SLIT3 deficiency promotes malignant potential and predicts a poor prognosis in NSCLC.

### 3.2 SLIT3 deficiency stimulated UBE2C upregulation

**Figure 2:**
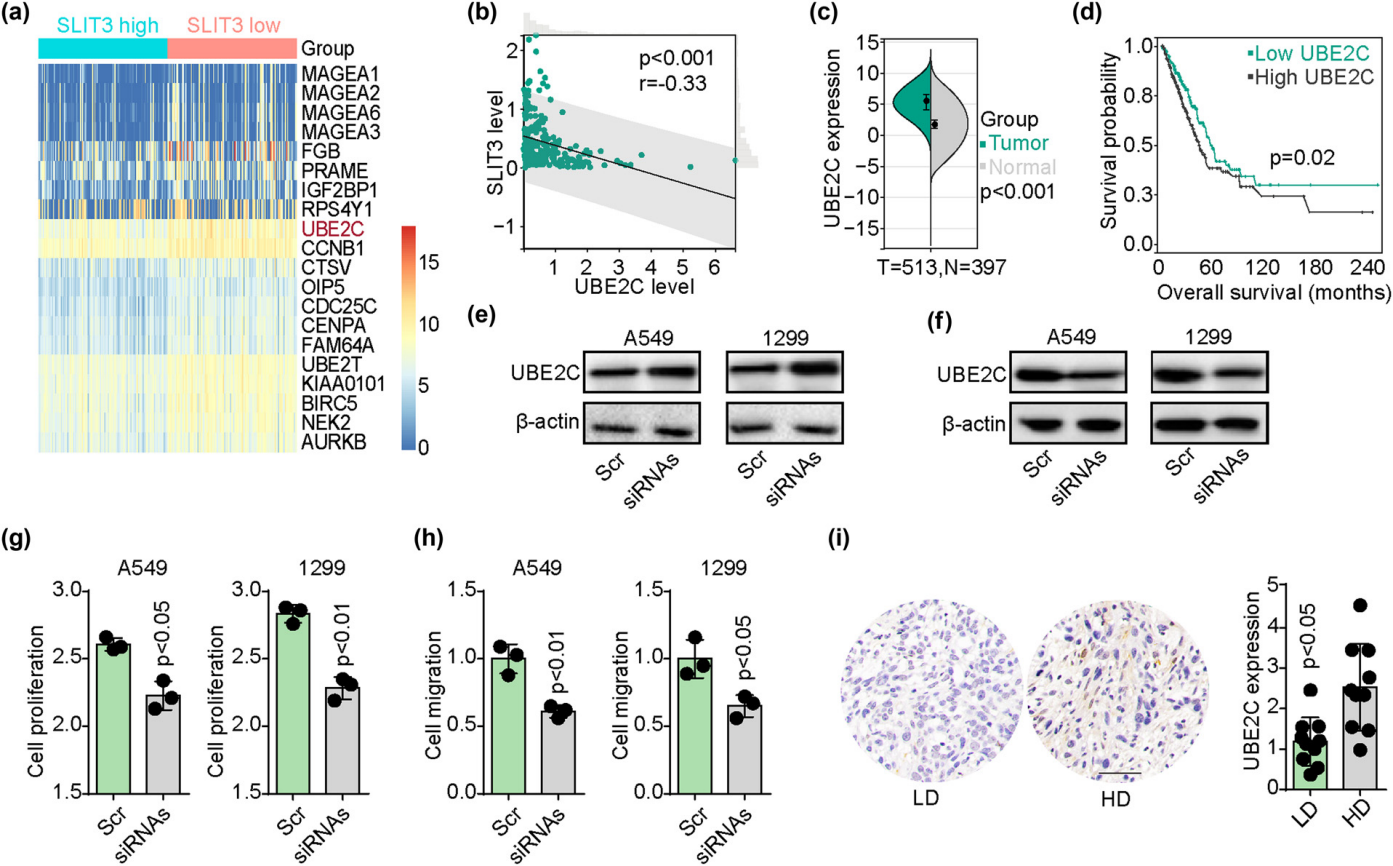
SLIT3 deficiency stimulated UBE2C upregulation. (a) 230 NSCLC patients from the TCGA database were categorized into SLIT3-high and -low groups, and the top 20 differentially expressed genes between these groups were enriched. (b) the correlation analysis between SLIT3 and UBE3C was conducted in 230 NSCLC patients. (c) mRNA expression of UBE2C in 513 NSCLC tissues and 397 normal tissues derived from TCGA data. (d) Kaplan-Meier overall survival curve was shown according to high and low expression of UBE2C in NSCLC patients (*n* = 513) derived from TCGA data. (e) Western blotting of UBE2C in A549/NCI-H1299 cells treated with scramble and SLIT3 siRNAs. (f) SLIT3 siRNAs treated A549/NCI-H1299 cells were treated with scramble and UBE2C siRNAs. Then the expression of UBE2C were examined by western blotting. (g) Cell proliferation of A549/NCI-H1299 cells in (f). (h) Cell migration of A549/NCI-H1299 cells in (f). (i) Immunostaining of UBE2C in NSCLC tissues from high-degree (HD, stage III–IV) and low-degree (LD, stage I–II) patients (*n* = 20). The scale bar was 100 μm.

Motivated by the findings that SLIT3 deficiency promoted the progression of NSCLC, this research aimed to elucidate the molecular pathway driven by SLIT3. To achieve this, 230 NSCLC patients from the TCGA database were categorized into SLIT3-high and SLIT3-low groups, and the top 20 differentially expressed genes between these groups were enriched (Figure 2a). Notably, UBE2C, belonging to the ubiquitin-conjugating enzyme (E2) family, is crucial for cell cycle progression and tumor development. The involvement of UBE2C in the progression of NSCLC was confirmed through correlation analysis between SLIT3 and UBE2C in the 230 NSCLC patients. Importantly, a negative correlation was observed between SLIT3 and UBE2C expression (Figure 2b). in addition, upregulated expression of UBE2C was noted in NSCLC tissues in comparison to normal tissues (Figure 2c). Furthermore, it was noted that patients with high UBE2C levels exhibited shortened survival times compared to those with low UBE2C levels (Figure 2d). These results suggest the possible role of UBE2C in SLIT3-related tumor progression. Subsequently, a western blotting assay was carried out to confirm the expression of UBE2C in SLIT3-silenced (or not) NSCLC cells. UBE2C was upregulated in SLIT3-silenced A549 and NCI-H1299 cells (Figure 2e). Furthermore, to evaluate whether UBE2C was involved in regulating progression of NSCLC, SLIT3-silenced A549/NCI-H1299 cells were treated with siRNA (UBE2C) for 48 h (Figure 2f). The cell proliferation (Figure 2g) and migration (Figure 2h) of NSCLC cells induced by SLIT3 interference were efficiently suppressed by the knockdown of UBE2C. The protein level of UBE2C was also upregulated in advanced-stage NSCLC patients (stage IV) compared to the low-stage group (stages I–III, Figure 2i). Collectively, these findings suggest that SLIT3 deficiency mediates UBE2C upregulation to promote NSCLC progression.

### 3.3 SLIT3/UBE2C axis modulated NSCLC progression through β-catenin/Wnt3A signaling

**Figure 3:**
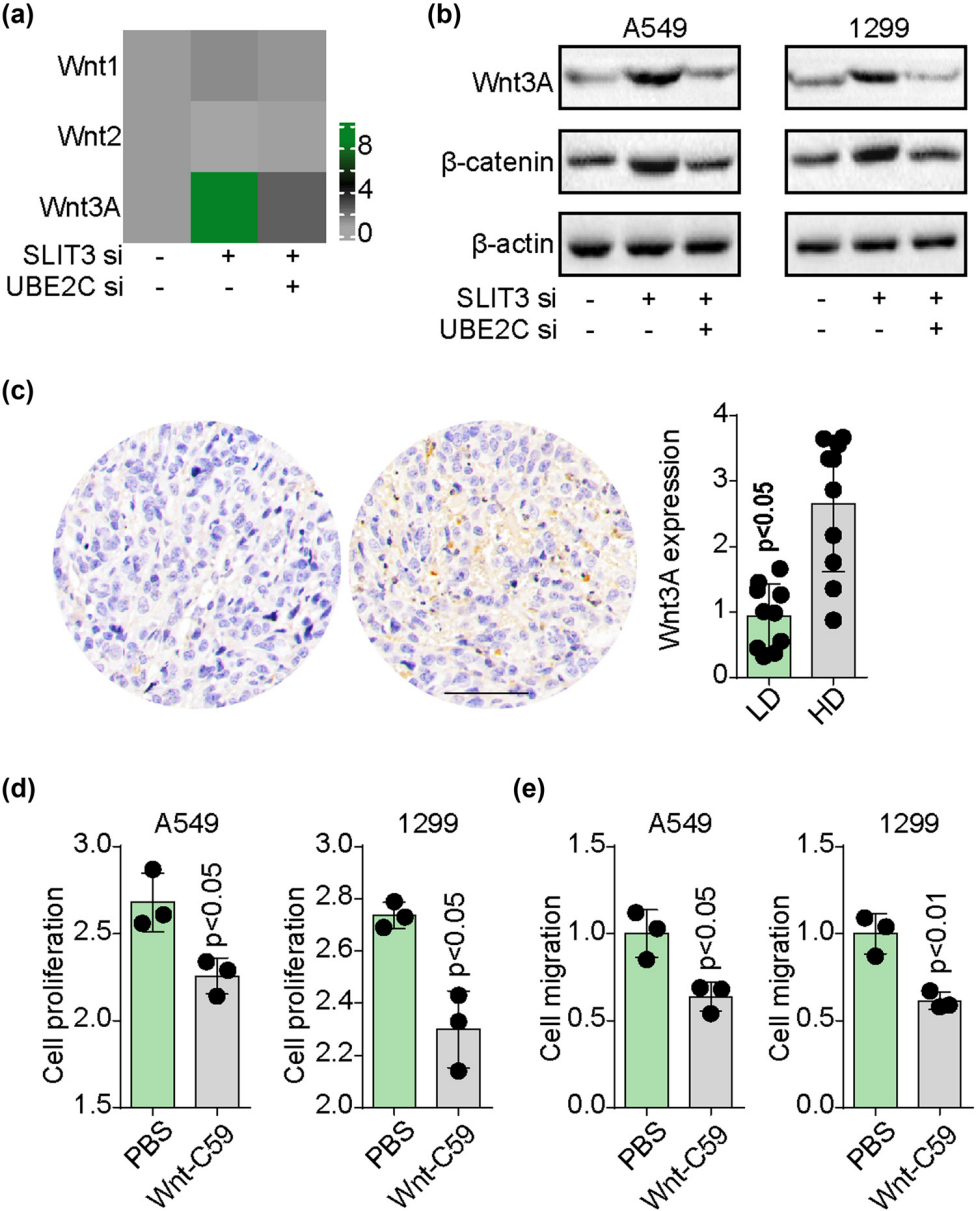
SLIT3/UBE2C axis modulated NSCLC progression through β-catenin/Wnt3A signaling. (a) the heatmap of Wnt1, Wnt2, and Wnt3A expression in A549 cells treated with scramble, UBE2C and SLIT3 siRNAs, respectively. (b) Western blotting of Wnt3A and β-catenin in A549/NCI-H1299 cells treated with scramble, UBE2C and SLIT3 siRNAs, respectively. (c) Immunostaining of Wnt3A in NSCLC tissues from high-degree (HD, stage III–IV) and low-degree (LD, stage I–II) patients (*n* = 20). The scale bar was 100 μm. (d) Cell proliferation of SLIT3 siRNAs pre-treated A549/NCI-H1299 cells, treated with PBS or Wnt-C59 (20 nM). (e) Cell migration of SLIT3 siRNAs pre-treated A549/NCI-H1299 cells, treated with PBS or Wnt-C59 (20 nM).

Increasing evidence suggests that UBE2C mediates tumor progression through the Wnt signaling pathway. To validate this hypothesis, the mRNA expression of Wnt1, Wnt2, and Wnt3A was examined in A549 and NCI-H1299 cells with silenced SLIT3 and UBE2C. Notably, silencing SLIT3 greatly upregulated Wnt3A, while siRNA for UBE2C suppressed this upregulation in both cell lines (Figure 3a). Similar results were observed at the protein level (Figure 3b), indicating that the SLIT3/UBE2C axis stimulated Wnt3A signaling activation in NSCLC cells. In addition, a high Wnt3A expression was observed in advanced-grade NSCLC tissues from patients (Figure 3c). Subsequently, SLIT3-silenced cells were exposed to Wnt-C59, a Wnt signaling inhibitor. Blockade of the Wnt signaling pathway effectively inhibited cell proliferation (Figure 3d) and migration (Figure 3e) in SLIT3-silenced NSCLC cells. These outcomes highlighted that the SLIT3/UBE2C axis mediates NSCLC progression through the Wnt3A signaling pathway.

### 3.4 Inhibition of Wnt signaling improved the outcome of chemotherapy

**Figure 4:**
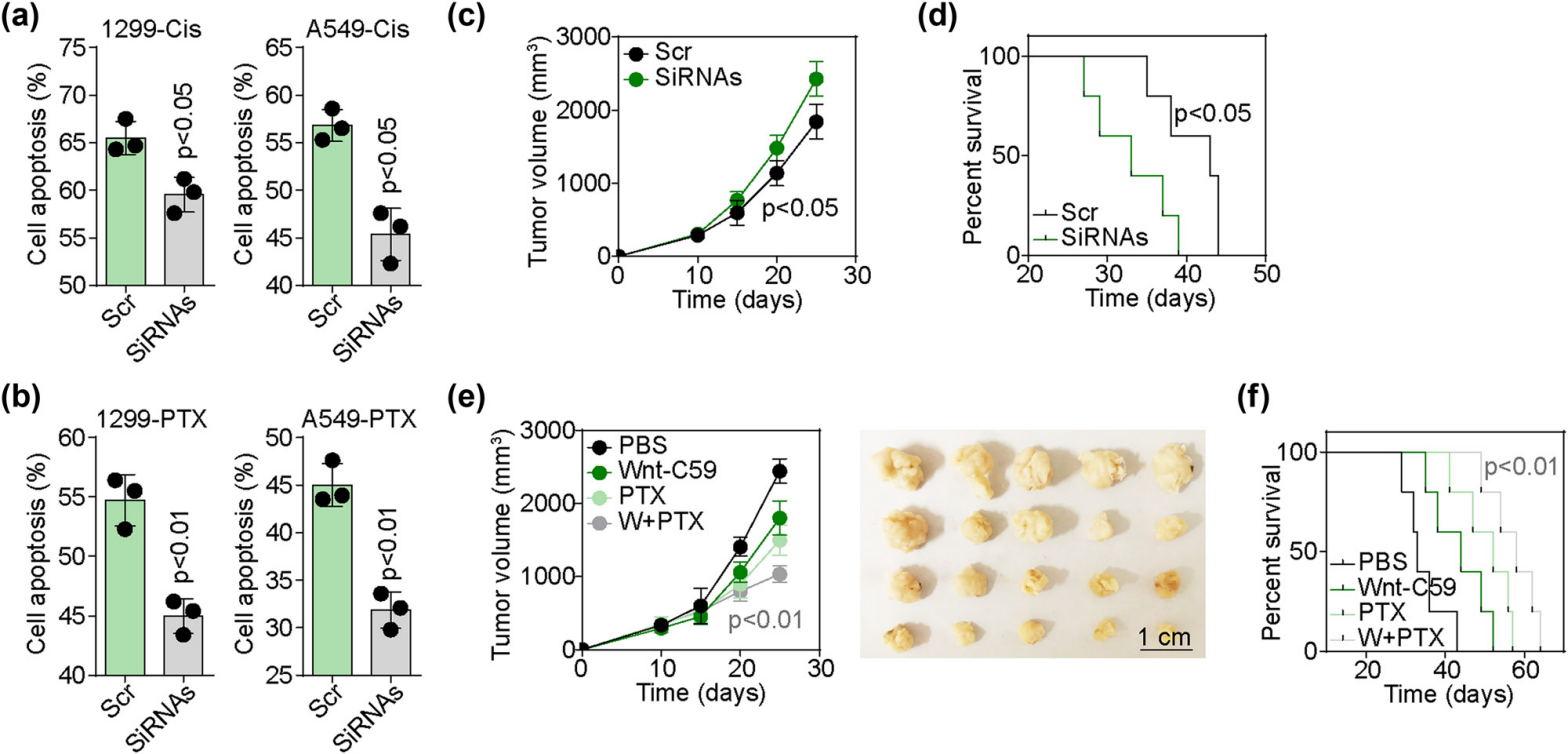
Inhibition of Wnt signaling improved the outcome of chemotherapy. (a) and (b) A549 and NCI-H1299 cells were pre-treated with scramble or SLIT3 siRNAs. Then cells were treated with Cis (1 μg/ml) or PTX (1 μg/ml) for 48 h. Cell apoptosis was examined. (c) and (d) A549 bearing mice (*n* = 5 in each group) were treated with scramble or SLIT3 siRNAs via intratumor injection on days 10, 15, and 20. Tumor volume and survival information were recorded. (e) and (f) SLIT3 siRNAs treated tumor-bearing mice (*n* = 5 in each group) were treated with PBS, PTX (10 mg/kg), Wnt-C59 (10 mg/kg), or a combination of them via tail vein injection on days 10, 15, and 20. Tumor volume and survival information were recorded.

The activation of the Wnt signaling pathway is closely linked to the development of chemoresistance in cancer. Therefore, A549/NCI-H1299 cells were exposed to chemotherapeutic agents (PTX and Cis) after silencing either scramble or SLIT3. The tumor cells with silenced SLIT3 showed increased resistance to PTX and Cis (Figure 4a and b). The impact of SLIT3 on NSCLC growth was assessed *in vivo* by establishing a subcutaneous A549-bearing mouse model with or without SLIT3 silencing and recording tumor volume and mouse survival. Consistent with our *in vitro* findings, A549-bearing mice treated with IT3 siRNAs exhibited a rapid tumor growth curve and shortened survival time compared to the scramble group (Figure 4c and d), indicating that the SLIT3/UBE2C/Wnt axis promotes NSCLC progression and chemoresistance. Due to the pro-tumor effects and chemoresistance development, chemotherapy was applied in combination with a Wnt inhibitor (Wnt-C59) to treat the A549-bearing mouse model with silenced SLIT3. As expected, Wnt-C59 significantly enhanced the tumor suppressive effects of PTX (Figure 4e) and prolonged the survival time of the tumor-bearing mice (Figure 4g). These experiments collectively suggest that the SLIT3/UBE2C/Wnt3A axis is crucially involved in promoting the progression of NSCLC, and that inhibition of Wnt signaling could improve the chemotherapy outcomes in NSCLC patients with SLIT3 deficiency.

## 4 Discussion

Lung cancer remains a substantial and persistent threat to human health on a global scale. Identifying the molecular mechanism and new targets is critically needed. In the course of this investigation, the impact of SLIT3 expression on the survival of lung cancer patients was assessed. Furthermore, the possible mechanisms by which SLIT3 might regulate lung cancer development by influencing the UBE2C expression and activating the Wnt signaling pathway activation in lung cancer cell lines were examined. Our study identified the tumor-suppressive role of SLIT3 in NSCLC. In addition, we have uncovered the molecular mechanism by which SLIT3 regulates NSCLC progression. Specifically, SLIT3 inhibits the UBE2C/Wnt signaling pathway, thereby regulating the occurrence and development of NSCLC.

SLITs (SLIT1-SLIT3), a family of secreted proteins, mainly mediates nervous system activity and function [18]. However, the recent work revealed that SLITs are also expressed in a series of malignancies, with potential roles in tumor occurrence and development [19,20]. Yang et al. [21] examined more abundant tumor lymph angiogenesis in SLIT2-overexpression transgenic mice, presenting with more tumor lymphatic metastasis. Zhou et al. [22] also implied that upregulated SLIT2 is linked to metastatic progression in colorectal carcinoma. However, the expression of SLITs is always repressed by DNA hypermethylation in multiple tumor types. Downregulated SLITs have been confirmed to promote tumor proliferation, migration, and invasion, which can lead to worse survival in lung cancer [23], breast cancer [14], and liver cancer [13]. In line with the previous study on SLITs in tumors, decreased SLIT3 was observed at the transcriptome and proteome levels in lung cancer tissues. It was noted that downregulated SLIT3 was related to advanced tumor stage and poorer prognosis. In addition, our study further demonstrates at the cellular level that SLIT3 inhibits the progression of NSCLC. Silencing SLIT3 expression played a tumor-promoting role in A549 and NCI-H1299 cell lines, suggesting that SLIT3 may act as a tumor suppressor gene in NSCLC. Moreover, we speculate that the inter-tumor heterogeneity, differential epigenetic gene regulation, and the tumor microenvironment may influence discordant functional states of SLITs, acting either as an oncogene or a tumor suppressor gene. Our study provides more substantial evidence directly proving the tumor-suppressive role of SLIT3 in the progression of NSCLC.

Previous studies have discussed SLITs binding to roundabout receptors (ROBO) as a crucial event in tumor progression. In breast cancer, loss of SLIT/ROBO signaling may decrease the phosphorylation of downstream CXCL12/CXCR4 molecules, increase GSK-3β phosphorylation, and influence the expression and distribution of β-catenin. All of these processes have been noted to contribute to pro-tumor activation [24]. However, the relevant mechanism of SLITs in lung cancer is still rarely reported. Here, 20 significantly differentially expressed genes were identified between SLIT3 high and low groups, and the *UBE2C* gene was set as the target gene for the subsequent study. UBE2C, a key component of the E2 ubiquitin-conjugating enzyme family, plays important roles in cell cycle processes and mitotic spindle checkpoint regulation [25]. Aberrant UBE2C expression disrupts the cell cycle progression and genomic instability, thereby leading to tumorigenesis. As previously reported, UBE2C was highly expressed in various human cancers and associated with adverse outcomes [26,27,28], which is consistent with our acquired results. Compared to previous studies, our research elucidates the regulatory role of SLIT3 and UBE2C. This investigation is the first to report that suppression of SLIT3 could upregulate the expression of UBE2C. By western blotting testing at a cellular level, we observed that depletion of SLIT3 resulted in increased expression of UBE2C. Furthermore, in tumor tissues from NSCLC patients, we identified a notable negative correlation between SLIT3 expression and UBE2C levels. Upregulated UBE2C exerts a synergistic efficacy with downregulated SLIT3 and is crucial in lung cancer progression. This finding provides preliminary evidence for the SLIT3/UBE2C axis that regulates tumor initiation and development in lung cancer.

Moreover, this research further verified that the SLIT3/UBE2C axis could mediate tumor cell proliferation and migration via Wnt3A/β-catenin signaling pathway activation. To our knowledge, abnormal activation of the canonical WNT/β-catenin signaling pathway is strongly linked to tumor onset, disease progression, and drug resistance [29]. These processes are involved in many tissue-specific and evolutionary WNT target genes, among which SLIT3 has been confirmed as a stomach-specific WNT target gene [30]. Other studies have described that UBE2C, along with the other signaling molecules such as AURKA, EMT, GRIK3, CDK1, and claudin 19, activates the WNT/β-catenin signaling pathway, thus altering tumor biology [31]. Similarly, in lung cancer, we observed downregulated SLIT3 and simultaneously upregulated WNT 3A expression, manifested by activating the WNT/β-catenin signaling pathway. In this process, the UBE2C gene functioned as a bridge. These results support the subtle association between the SLIT3/UBE2C axis and the WNT/β-catenin signaling pathway in lung cancer. The data acquired in this investigation provide an important addition to the mechanism for lung cancer progression. Moreover, we argue that SLIT3 may be a common but not tissue-specific target gene of WNT. However, this requires further validation in other cancers. This research demonstrated the harmful effect of a hyperactivated WNT/β-catenin pathway on the response to chemotherapy in a mouse model, consistent with a prior report [32]. However, our study has some limitations. First, the number of clinical samples is small (20 cases). Future research should include a larger cohort of clinical patients to explore the correlation between the SLIT3/UBE2C axis and NSCLC. In addition, the direct interaction between SLIT3 and UBE2C remains unclear. It is necessary to investigate whether SLIT3 affects UBE2C through the regulation of common signaling pathways or cellular processes. For instance, SLIT3 and UBE2C might be regulated by shared signaling pathways such as the PI3K/Akt pathway, or their gene expressions could be influenced by common upstream or downstream regulatory factors such as transcription factors or microRNAs. Further studies are required to elucidate the specific molecular mechanisms involved.

## 5 Conclusions

The current study elucidated that SLIT3 expression was downregulated *in vitro* lung cancer cell assays and related to poor prognosis. Further, the data acquired in this research presented the preliminary proof for SLIT3/UBE2C/WNT signaling pathway on lung cancer development and progression, providing novel insight and strategy in clinical NSCLC treatment.
